# Convolutional neural network based data interpretable framework for Alzheimer’s treatment planning

**DOI:** 10.1186/s42492-024-00154-x

**Published:** 2024-02-01

**Authors:** Sazia Parvin, Sonia Farhana Nimmy, Md Sarwar Kamal

**Affiliations:** 1 Information Technology, Melbourne Polytechnic, Melbourne, VIC 3072 Australia; 2 Faculty of Economics and Business, University of New South Wales, Sydney, ACT 2612 Australia; 3 School of Computer Science, Faculty of Engineering and IT, University of Technology Sydney, Sydney, NSW 2007 Australia

**Keywords:** Multimodal, Region-based convolutional neural network, Layer-wise relevance propagation, Submodular pick local interpretable model-agnostic explanations, Graphical genes tree, Alzheimer’s disease

## Abstract

Alzheimer’s disease (AD) is a neurological disorder that predominantly affects the brain. In the coming years, it is expected to spread rapidly, with limited progress in diagnostic techniques. Various machine learning (ML) and artificial intelligence (AI) algorithms have been employed to detect AD using single-modality data. However, recent developments in ML have enabled the application of these methods to multiple data sources and input modalities for AD prediction. In this study, we developed a framework that utilizes multimodal data (tabular data, magnetic resonance imaging (MRI) images, and genetic information) to classify AD. As part of the pre-processing phase, we generated a knowledge graph from the tabular data and MRI images. We employed graph neural networks for knowledge graph creation, and region-based convolutional neural network approach for image-to-knowledge graph generation. Additionally, we integrated various explainable AI (XAI) techniques to interpret and elucidate the prediction outcomes derived from multimodal data. Layer-wise relevance propagation was used to explain the layer-wise outcomes in the MRI images. We also incorporated submodular pick local interpretable model-agnostic explanations to interpret the decision-making process based on the tabular data provided. Genetic expression values play a crucial role in AD analysis. We used a graphical gene tree to identify genes associated with the disease. Moreover, a dashboard was designed to display XAI outcomes, enabling experts and medical professionals to easily comprehend the prediction results.

## Introduction

In healthcare systems and clinical practice, an array of artificial intelligence (AI) tools and machine learning (ML) methods has gained popularity among doctors and researchers [1]. The application of ML algorithms in AI-driven health diagnostics has proven to be efficient for early detection and personalized treatment recommendations. However, it is essential to consider multiple data sources and mats to enhance clinical efficiency and achieve accurate outcomes from patient health data. Through the synergistic integration of AI and ML in healthcare, we can unlock the unprecedented potential to revolutionize medical decision-making, improve patient outcomes, and transform the modern medical landscape.

Researchers have recently turned their attention to multimodal data, because clinical and healthcare administrators require the analysis of complex decision-making outcomes across diverse data formats [2–4].

Typically, doctors and experts rely on a wide range of data formats for patient healthcare records, such as image data (e.g., magnetic resonance imaging (MRI), X-rays, photographs, and computerized tomography scans), tabular data (e.g., demographics, medical history, and age), and genetic information (e.g., gene expression, protein expression, and molecular functionalities) [5–9].

This prompted us to develop a multimodal data framework for Alzheimer’s disease (AD) analysis. AD is a neurological disorder that impairs the human brain [10–12]. AD is the most common type of dementia, which results in changes in normal behavior, memory shortages, and a decline in thinking capabilities. This disease is characterized by the abnormal accumulation of amyloid plaques and neurofibrillary tangles in the brain in multiple stages. Many studies have been conducted to detect the progression of this disease and identify effective diagnostic methods. Alberdi et al. [13] predicted that 11 million to 16 million elderly people are likely to suffer from AD by 2050, whereas 7 million patients are already infected with AD in the United States as of 2022. Early and accurate diagnosis can help mitigate primary brain damage. As AD does not have any effective recovery, early detection through multimodal data analysis could be considered a proactive and timely treatment that can delay the progression of this disease. Early detection has been considered an important step in the development of advanced treatments for AD [14]. A comprehensive literature review was conducted by concentrating on patient datasets and effective measurement techniques, including ML. Two ML methods, an 18-layer convolutional network, and a 3D convolutional network, were employed to forecast the research outcome. Contemporary medical tools and healthcare systems can significantly enhance patient outcomes [15–18].

Owing to the prevalence and severity of the disease, current diagnostic tests often struggle to provide a detailed understanding or definitive results within a patient’s lifetime, relying heavily on a comprehensive analysis of the patient’s medical history and information. Examining brain tissue changes can aid in the most accurate AD diagnosis; however, collecting samples through biopsies poses high risks to patients [19–22]. Neurological changes induced by AD can be effectively diagnosed using MRI and ML. Deep-learning techniques (deep convolution networks) have been applied to analyze medical images to detect abnormalities, classify diseases, and diagnose diseases [23]. Different data decision-making processes can be applied to improve and extend the accuracy and efficiency of medical image analysis using deep convolution networks, thereby strengthening patient care. Computer vision and deep learning (DL) methods have been effectively used in ref. [24] to accurately detect AD, with an accuracy of 97.65%. Convolutional neural network (CNN) has been used in DL through meaningful optimization of precious experiences using eight later architectures in this particular work. Modern healthcare and the use of DL models for early AD detection using neuroimaging biomarkers have presented significant challenges in AD. Researchers have implemented an EfficientNet-b0 CNN with a novel “fusion of end-to-end and transfer learning” approach to classify different stages of AD [25].

In addition to the histological examination of MRI images, the accurate diagnosis of AD severity depends on other data sources, such as demographic and gene expression data. Genetic information plays a significant role in the diagnosis of AD. Furthermore, a strong correlation has been observed between gene expression data and patient demographic information for the diagnosis of AD, leading to improved treatment for AD patients [26–28]. Consequently, recent research has shown a growing interest in utilizing multimodal data to detect different acute diseases. This study aimed to determine the severity of AD using multimodal data analysis.

Although multimodal data analysis using various ML models has attracted the attention of researchers, these ML models, often referred to as “black box” models, can be challenging to fully comprehend. Explainable artificial intelligence (XAI) approaches can deliver reliable and trustworthy medical and clinical data by offering insights into prediction models. To interpret image datasets, several XAI techniques, such as gradient-weighted class activation mapping, layer-wise relevance propagation (LRP), and concept activation vectors, are employed to explain CNN for glaucoma prediction from MRI images [29, 30]. Additionally, XAI approaches such as local interpretable model-agnostic explanations (LIME) and Shapley additive explanations (SHAP) are used to explain tabular or demographic data.

In this study, we focused on multimodal data to predict AD and enhance the explainability and interpretability of prediction models. To process the MRI data, we generated a knowledge graph from the image and applied a CNN to predict the severity of AD. CNN is a black box method for identifying patients with or without dementia. To increase the reliability of the results predicted by the CNN, we employed LRP. The LRP approach explains and interprets CNN results. To analyze AD from the medical records (tabular data), we used a support vector machine (SVM) to classify patients with AD. However, SVM is a black box model; therefore, we utilized LIME as the XAI approach to interpret the prediction insights for obtaining interpretable outputs. LIME helps identify demographic features that significantly contribute to AD. We also applied a XAI approach called the graphical gene tree (GGT) to interpret the gene expression data. GGT aids in identifying the genes associated with AD.

This research presents two significant results of practical importance.

1. Enhanced decision-making for personalized treatment: One of the crucial necessities of this research is to empower healthcare professionals to make better-informed decisions regarding personalized treatment plans for Alzheimer’s patients. By incorporating an interpretable framework using multimodal data, doctors and researchers can gain a deeper understanding of the complex factors contributing to the disease, including genetic, demographic, and imaging data. This comprehensive knowledge enables them to tailor treatments to individual patient needs, ultimately improving patient outcomes and quality of life.
2. Encouraging collaborative healthcare innovation: The development and application of an interpretable framework for Alzheimer’s treatment planning using multimodal data can foster collaboration among different stakeholders in the healthcare ecosystem, including researchers, clinicians, data scientists, and technology developers. Interdisciplinary collaboration encourages the exchange of knowledge, expertise, and resources, ultimately promoting the development of advanced, effective, and accessible healthcare solutions. Such collaborative efforts can contribute to better healthcare outcomes and drive positive societal change by ensuring that patients with AD receive the best possible care irrespective of their socioeconomic background.

## Methods

In the methodology for an interpretable framework for multimodal data analysis, we first preprocessed and cleaned data from various modalities, such as tabular, image, text, and gene expression data. Next, we integrated the data by aligning and connecting features from different sources to create a unified dataset. To combine the features of these multimodal data, we first used MRI to identify brain regions affected by AD and the genes responsible for these changes. We then identified the corresponding genes for different stages of AD, including mild dementia, moderate dementia, non-dementia, and very mild dementia. We then applied black box AI models to each modality considering the specific characteristics of the data type. To enhance the interpretability of the results, we utilized XAI techniques tailored to each modality, allowing for a better understanding of the model’s predictions. Finally, we evaluate the framework’s performance using appropriate metrics to assess the accuracy and interpretability of the multimodal data analysis.

### Datasets

In this study, we analyzed AD using multimodal data. This multimodal dataset is open access and comprises three different modalities (tabular, image, and gene expression data) from four distinct data sources. The open access series of imaging studies (OASIS) dataset created by the Washington University Alzheimer’s Disease Research Center contains patient medical information. These medical records were obtained from Kaggle (medical record). The OASIS dataset includes information on 416 patients aged 18–96 years categorized into three different years (young, middle-aged, or older adults). This dataset contains MRI scans of 150 patients aged 60–96 years obtained over two or more visits at least one year apart. Each participant was scanned three to four times during each session. All participants were right-handed and included both men and women. Seventy-two people had no dementia throughout the study, while 64 had dementia at their first visit and remained, including 51 with mild to moderate AD, and the rest aged 18–59 years. To analyze the severity of AD, 64000 MRI images were sourced from Kaggle (image data), comprising image data that included four different Alzheimer’s categories: mild dementia, moderate dementia, non-dementia, and very mild dementia. Each image was derived from the aggregation of three or four separate T1-weighted MRI scans of both male and female subjects. Microarray data were obtained from the NCBI Center for Biotechnology Information (accession no. GSE174367), which contains 18234 genes in rows and 104 patients in columns representing either AD or non-AD cases [31, 32].

### Patient-centric multimodal data architecture

**Fig. 1:**
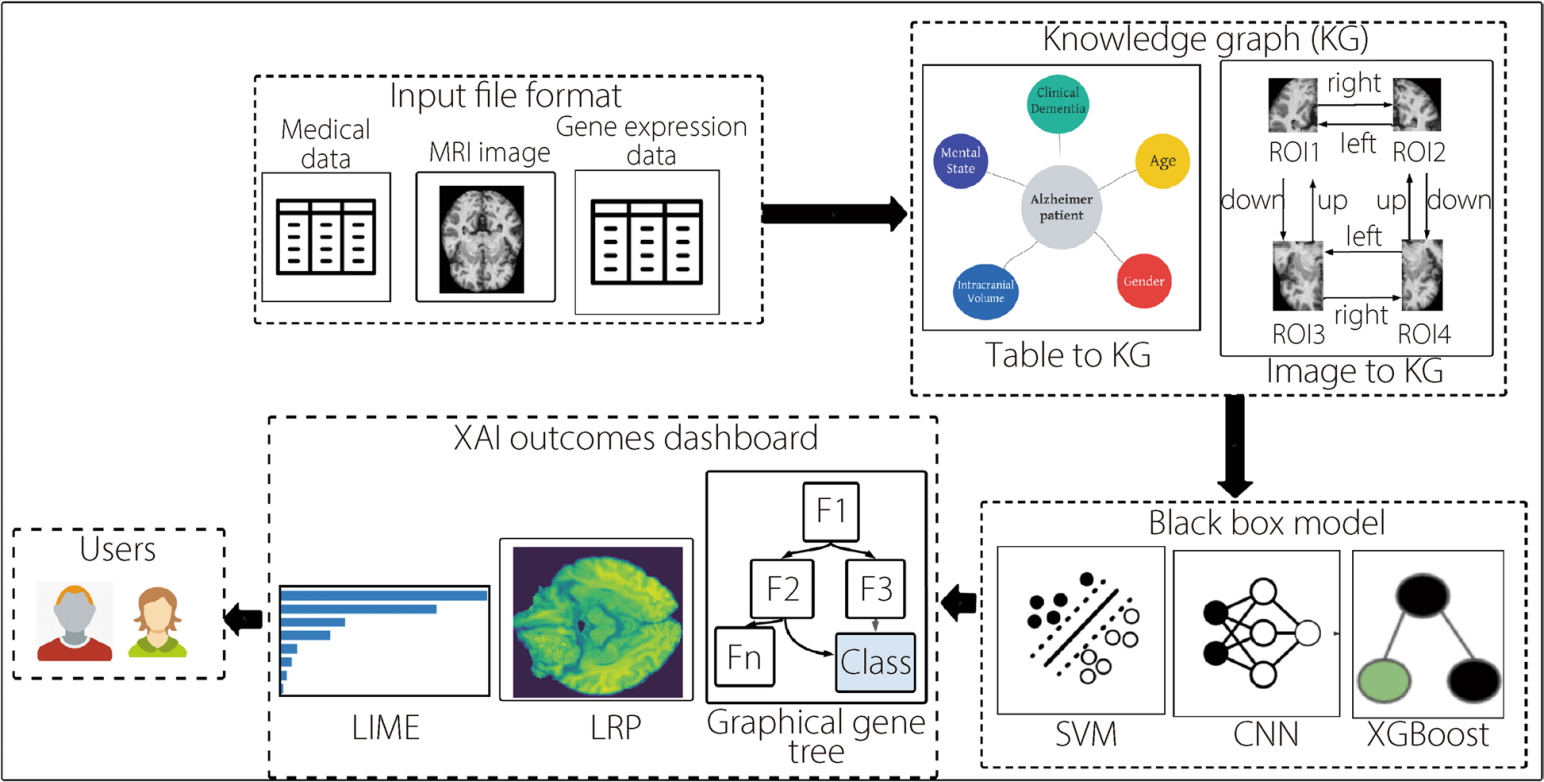
A schematic diagram for interpretable multimodal data analysis

In this study, we present a method for patient-specific multimodal data explainability for AD comprising of three stages: data pre-processing, knowledge graph generation, and data explainability and interpretability. During the data pre-processing phase, we processed the collected multimodal datasets. Then, we applied various knowledge graph generation approaches (for example, Image2Graph and Text2Graph) to the preprocessed data to create knowledge graphs. XAI techniques, such as LIME, SHAP, local interpretation-driven abstract Bayesian network, and LRP were used to explain the multimodal datasets for AD (Fig. 1).

### Knowledge graph

A knowledge graph [33] is a directed graph characterized by entity categories and descriptions. It is defined as a tuple, *G* = *E, R, T, C, D*, where *E* represents a set of entities, *R* denotes the relationships between entities, *T* is a set of triples, *C* signifies the entity categories, and *D* refers to the set of entity descriptions. A single tuple, *t* ∈ *T*, takes the form of (*e*_*i*_*, r*_*j*_*, e*_*k*_), where *e*_*i*_*, e*_*k*_ ∈ *E* are the top and bottom entities, and *r*_*j*_ represents the relationship between them.

### Knowledge graphs from tabular data

**Fig. 2:**
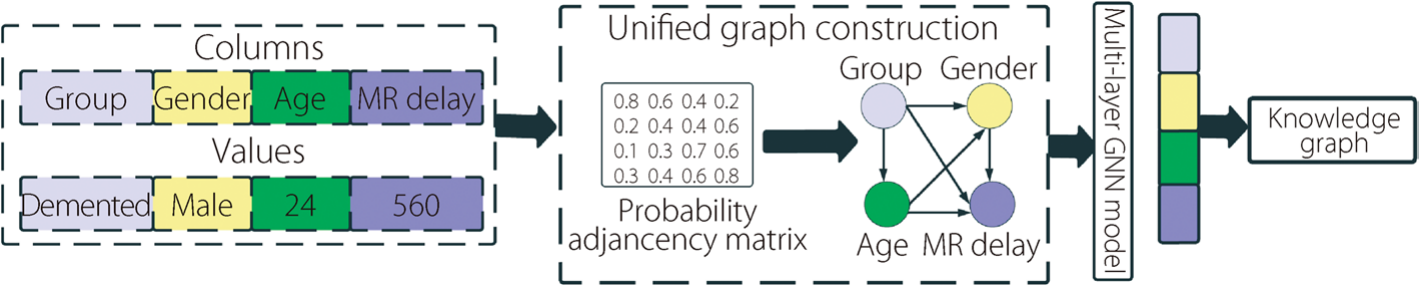
A schematic diagram to generate knowledge graphs from tabular data

A model that converts tabular data into a knowledge graph by extracting features from it was discussed. As shown in Fig. 2, we employ a graph neural network (GNN) to generate a knowledge graph.

#### Graph construction with probability adjacency matrix

Given *m* columns in tabular data, denoted as $x=\{{x}_{1},{x}_{2},\dots {x}_{m}\}$ , we represent these columns as an embedding matrix, $E\in {R}^{m\times d}$, to construct a unified graph. Each column *x*_*j*_ is embedded in the j-th row of the embedding matrix E. We compute the probability adjacency matrix, *A*, using the following equation

1 $$A =\mathrm{ softmax}\left({\sigma ({EW}_{l})\sigma ({EW}_{r})}^{T}\right){\in R}^{m\times m}$$

Here, ${W}_{l},{W}_{r}\in {R}^{m \times m}$ are trainable matrices, *σ* is the activation function, and the *sigmoid* function normalizes the link weights among table columns.

#### Feature interaction learning

Considering a row for a sample, denoted as ${x}^{i}=\left\{{x}_{1}^{i},{x}_{2}^{i},\dots {x}_{m}^{i}\right\}$ , we transform these features into a feature embedding matrix, $\in {R}^{m\times d}$E, where each row represents the features of a sample. This embedding matrix E was used to initialize the nodes in the knowledge graph.

We then used a GNN approach to learn feature interactions in the tabular data. The GNN recursively updates the node-embedding values for each node by using different internal layers. The k-th convolution layer of the GNN is defined as:

2 $${Ek}^{i}={Ek}^{0} + \sigma \left(AEk-{1}^{i}Wk\right)$$

${Ek}^{i}\in {R}^{m \times d}$ is the intermediate embedding feature matrix, E0^*i*^ is the initial embedding matrix, and ${W}_{k}\in {R}^{d \times d}$ is a trainable matrix. The GNN aggregates intermediate neighborhood information from the initial embedding matrix, E_0_.

#### Node link sampling

We also describe a method for generating the weights of the links among the nodes. The number of feature interactions for each row can be defined as

3 $$Li=RowSample(E[i,:],s)=\{(i,j1),....(i,js)\}$$

Here, *s* is the sample size, and the RowSample function determines link weights based on the multinomial probability distribution L=U.

### Knowledge graphs from images data

**Fig. 3:**
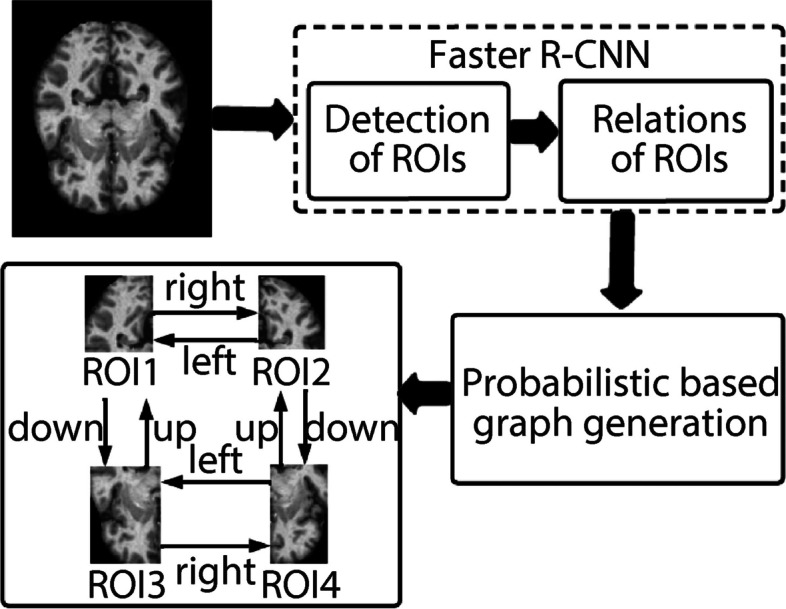
A framework to generate knowledge graphs from Alzheimer’s MRI data

The generation of a knowledge graph from image data, which demonstrates the interconnections between different brain regions in AD MRI data, is also discussed. As depicted in Fig. 3, the knowledge graph generation framework involves three primary steps: detecting regions of interests (ROIs), determining the relationships among ROIs, and creating graphs.

#### Identifications of ROI

We employed a faster region-based convolutional neural network (R-CNN) model to identify ROIs within the faster R-CNN images [34]. Initially, a complete MRI image is processed using a CNN [35], generating features specific to the given MRI image. Subsequently, these image features were passed through another neural network, the region proposal network [36], which predicts the ROIs for the corresponding image along with the associated bounding boxes. By mapping these ROIs with image features, we can extract specific regions from the MRI images based on the identified bounding boxes.

#### Relations between the ROIs

We explore the relationships between the ROIs that are crucial for constructing the graph. The extracted ROIs were processed using an R-CNN to establish connections among them.

Mathematically, the knowledge graph generation process entails estimating the optimal ${y}^{*}={max}_{y}P\left(y|I,{B}_{I}\right)$ that maximizes the following probability function:

4 $$P\left(\left(y|I,{B}_{I}\right)=\right)\prod\nolimits_{i\in V}\prod\nolimits_{j\ne i}P\left({y}_{i}^{cls},{y}_{i}^{bbox},{y}_{i\to j}|I,{B}_{I}\right)$$

*I* denotes an MRI image, B_I_ represents the proposed object boxes, and *y* is a set of all variables, including classes (demented and non-demented), bounding boxes, and relationships:${y=\{y}_{i}^{cls},{y}_{i}^{bbox},{y}_{i\to j}|i=1,\dots .n,j=1,\dots .,j\}$. Here, *n* refers to the number of proposed boxes, *y*_*cls*_ indicates the class label, and *y*_*i*→*j*_ represents the predicate between the i-th and j-th proposed boxes. We selected the regions associated with the highest probability values, $P\left(y|I,{B}_{I}\right),$ for the bounding boxes.

#### Construction of knowledge graph

The extracted probability values enabled the construction of a knowledge graph. To achieve this, we considered a graph with triples.

5 $$\prod G=\left\{\left(x,Px,\alpha \right):\prod x|{P}_{x}=\alpha \right\}$$

where *x* represents an ROI of image *I*, *P*_*x*_ is the Cartesian product of the ROI probability values, and *α* denotes the connected weight among the ROIs.

Ultimately, the knowledge graph can be defined as:

6 $$\sum =\{(\neg {{x}_{i}}{\vee}{p}_{xi},1-{\alpha }_{i})/({\alpha }_{i},{P}_{x},\alpha )\epsilon {\Pi }_{G}\}$$

#### Explainable AI Methods for Healthcare Systems

Several healthcare systems based on ML and multimodal data are viewed as black boxes because of their explainability and interpretability. To achieve trustworthy and interpretable results, we employed various XAI approaches to interpret our findings.

### GGT approach for XAI

**Fig. 4:**
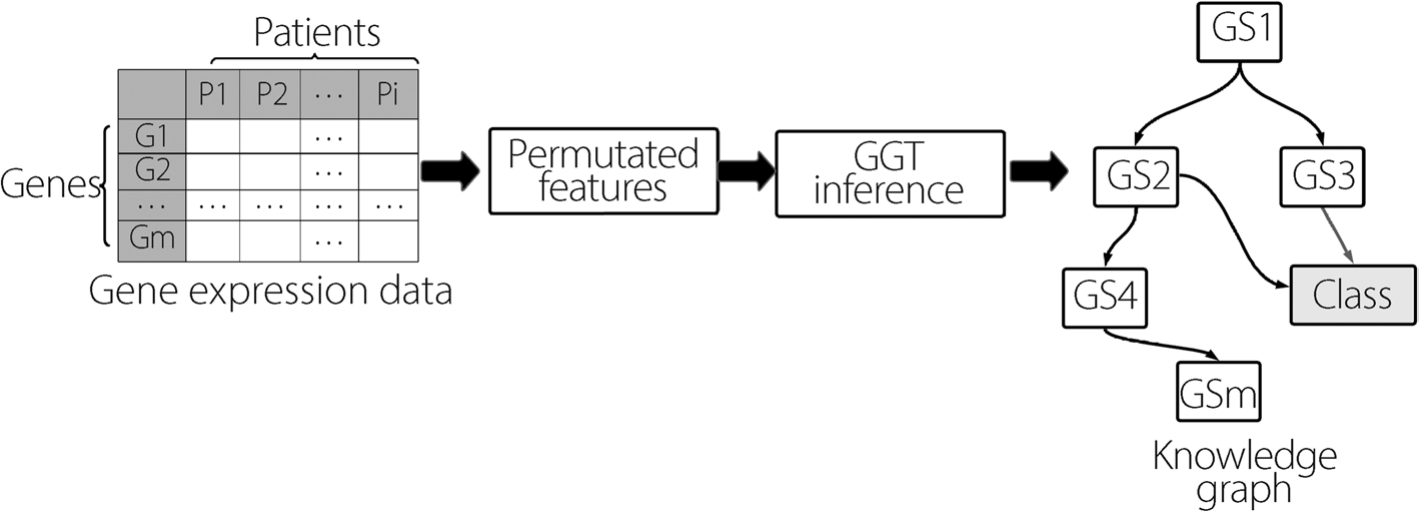
An overall framework to generate knowledge graphs from Alzheimer’s MRI data

This subsection outlines the XAI approach used to identify genes relevant to AD using gene expression data. We applied the GGT method to extract biological knowledge related to AD. The GGT is an interpretable approach that helps explain the predictive mechanisms of ML and generates knowledge graphs. The GGT framework, depicted in Fig. 4, comprises three basic steps: (1) permutation generation, (2) Bayesian network learning, and (3) breadth-first search (BFS) to find class variables.

### Permutation generation

GGT permutes the vector of feature inputs (genes) with *F = {F*_*1*_*, F*_*2*_*, …, F*_*n*_*}*. Features are permuted using a uniform distribution with permutation variance, *ϵ*, where ϵ ε [0,1]. This permutation is performed over the interval *F*_*i*_ − *ϵ, F*_*i*_ + *ϵ*. The goal of the permutation is to investigate how permutations affect the prediction of classifiers for different combinations.

### Bayesian network learning

During the Bayesian network learning phase, GGT creates a Bayesian network. A Bayesian network is a directed acyclic graph in which each node represents a variable and each edge represents the direct connectivity from the source node to the goal node. Bayesian networks represent the dependency/independence between features, and each node is associated with a conditional probability [37]. Bayesian networks calculate the probability chain rule in full-join probability theory [38].

Let G be a BN graph of features *F*_1_*, F*_2_*, …, F*_*n*_. The probability of exceeding P over the sample for graph G can be expressed using the following equation [39]:

7 $$P\left({F}_{1},{F}_{2},\dots ,{F}_{n}\right)=\prod\nolimits_{i=1}^{n}P({F}_{i}|{P}_{aFi})$$

Here, *P*_*aFi*_ represents all the parent variables for feature *F*_*i*_. Bayesian networks work together with all variables using full joint probability theory for inference.

This Bayesian network has two important parameters: a directed acyclic graph *G* and a set of conditional probability parameters *ϕ* representing the conditional dependency. Given gene expression data *d* with *n* observations, *P* (*G, ϕ d*) comprises two phases: structure learning and parameter learning, as described below [40]:

8 $$P\left(G,\upphi |d\right)=p\left(G|d\right).P\left(\upphi |G,d\right)$$

Here, *p* $\left(G|d\right)$ is structure learning, and *P* $\left(\upphi |G,d\right)$ is parameter learning. Structural learning aims to determine a directed acyclic graph G by maximizing P $\left(G|d\right)$. Parameter learning focuses on the probability parameter learning focuses on the probability parameter *ϕ* obtained from structural learning.

Given the parameter *ϕ* with an independent distribution, the learning process can be described as follows [41, 42]:

9 $$P\left(\upphi |G,d\right)={\prod }_{i}P\left({\upphi }_{{F}_{i}}|\prod {F}_{i},d\right)$$

#### Class variable searching

GGT uses a BFS, a feature selection approach for a specific class. The BFS identifies the target variables from the parents, child, and parent (parent of a child) of the target variable. GGT identifies interconnected genes that are directly associated with the target variables.

### XAI approach: LRP

Next, we introduce LRP [43] to explain the outcomes of the CNN approach for AD class prediction from image data. The main idea of the LRP algorithm is to compute the relevance score of the features for individual MRI images and track the contribution of the final output through layer-by-layer operations. In the LRP algorithm, each node in layer *l* contributes to the activation node *j* in the immediate following layer *l* + 1 receives a relevance score ${R}_{l+1}^{j}$.

The total relevance score of layer *l* was determined by summing all the relevance scores for neuron *i*. The overall relevance score can be defined as

10 $$\sum\nolimits_{i}{R}_{l,l+1}^{i\to j}={R}_{l+1}^{i}$$

Here, $\sum_{i}{R}_{l,l+1}^{i\to j}$ is the overall relevance score.

#### Explainability using submodular pick-LIME

To demonstrate the interpretability of tabular data from patients with AD, an experiment was developed using the LIME approach with the variant called submodular pick LIME (SP-LIME) [44], which shows how a particular decision is made concerning the associated medical features. SP-LIME is a global interpretation model and extended framework of the LIME process. The SP-LIME formalism is as follows:

Let *X* be the space of Alzheimer’s patients’ medical features and *x* be an instance of tabular data. LIME was used to explain the predictive models. LIME has two main components: explanation (f) and black box model (p). For this explanation, LIME uses an interpretable function as follows (Eq. 11):

11 $${\text{exp}}\left(x\right)={argmin}_{f\in F}\theta \left(p,f,{\lambda }_{x}\right)+\Omega (f)$$

Where *exp*(*x*) represents the interpretable features explained by LIME, the loss function *θ*(*p, f, λ*_*x*_), *p* denotes the black box model (i.e., decision tree), *f* signifies the explanator, and *λ*_*x*_ is the similarity measure between data points *x*. The penalty for the complexity of model *f* is represented by Ω. We solved Eq. 12 using the provided HR data, and LIME locally explained the job satisfaction characteristics.

The feature set ***V*** is defined by SP-LIME for the entirety as follows:

12 $$C\left(V,W,I\right)=\sum\nolimits_{j=1}^{d}\left[\exists i\in V:{W}_{ij}\right]{I}_{j}$$

Here, *B* is the total number of explanations that the user is willing to consider, *W* is the explanation matrix on *n* × *d*, where *n* is the sample size and *d* is the set of patient medical features. The global importance across the explanation space is denoted by *I*_*j*_, *V* represents the features that are explained, and *C*(*V, W, I*) is the overall importance rating of the features.

## Results and Discussion

In this section, we present the outcomes of AD analysis in terms of explainability and interpretability for multimodal data analysis. First, we describe the XAI outcomes for AD medical records, followed by subsections addressing the XAI outcome analysis of images and gene expression data. We combined all XAI outcomes on a dashboard for doctors and experts.

**Fig. 5:**
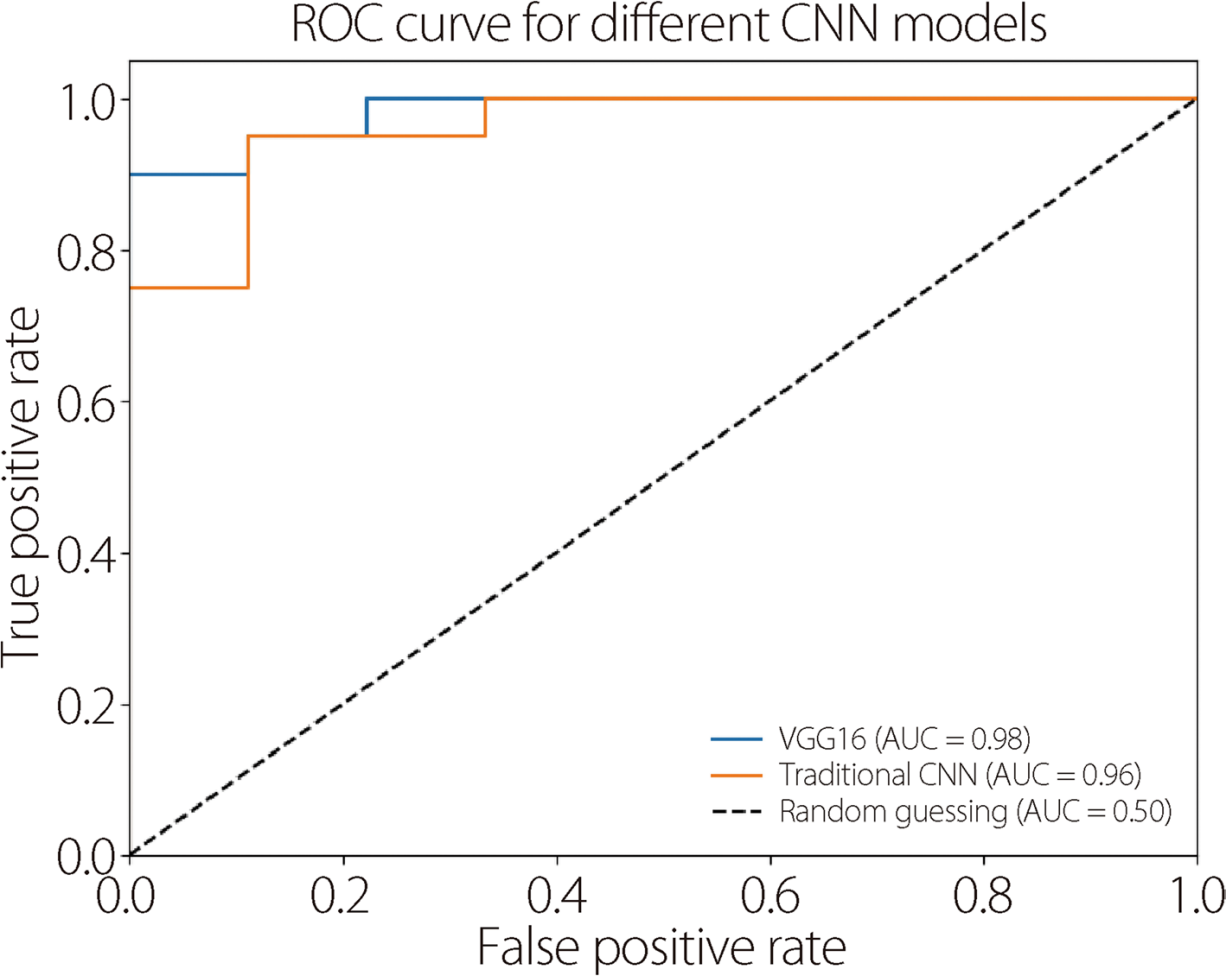
ROC curve to compare the predicted outcomes between two CNN models using MRI images

**Fig. 6:**
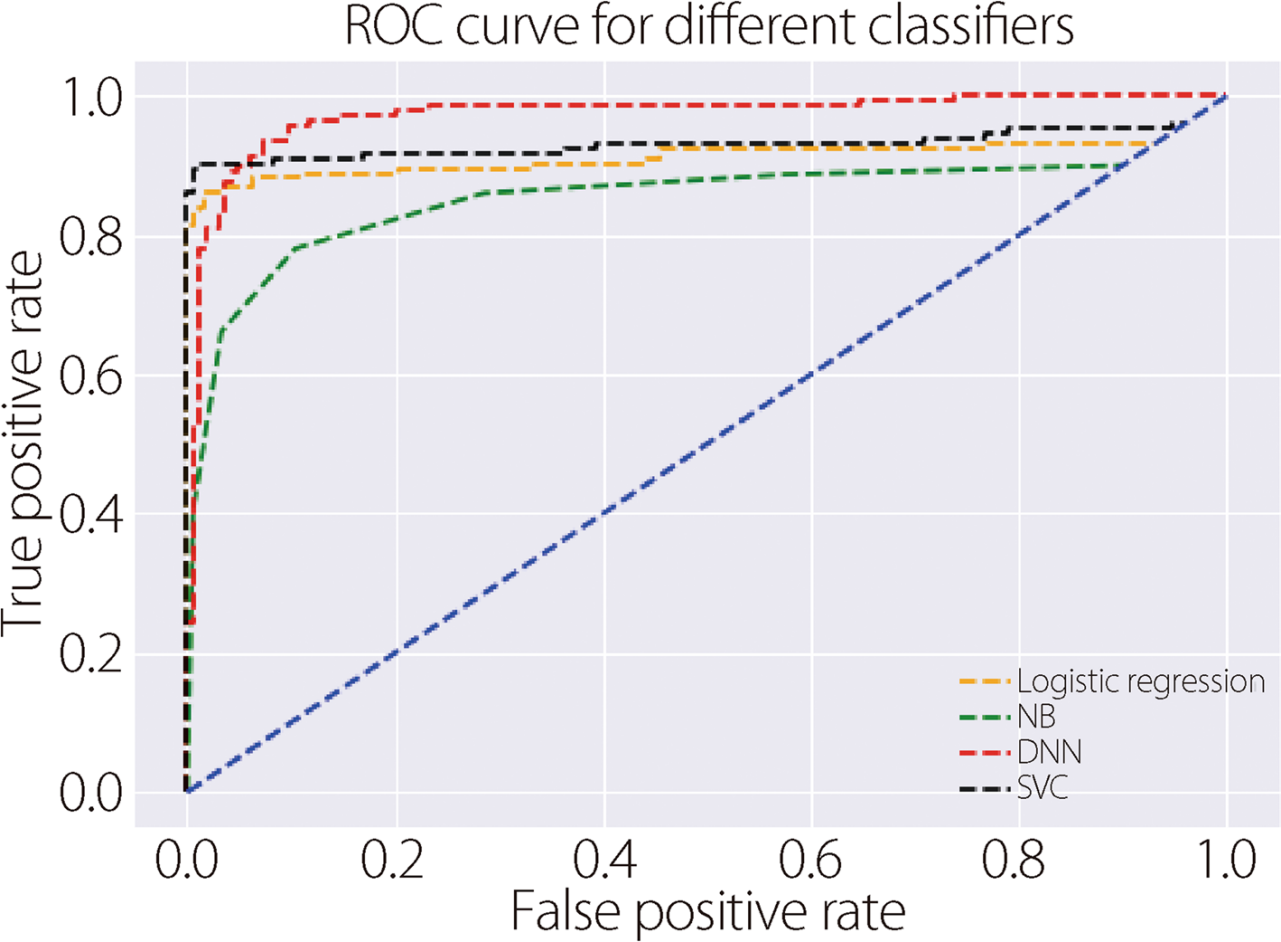
ROC curve to compare the predicted outcomes between different classifiers using gene expression data

We compared traditional CNN [25] and VGG16 models for disease identification. We plotted receiver operating characteristic (ROC) curves and calculated the ROC area under the curve (AUC) for both models (Fig. 5). This shows how well each model distinguished between AD and non-AD samples at different decision thresholds. We found that VGG16 (AUC: 0.98) was more accurate than the traditional CNN (AUC: 0.96), because it is deeper, has more features in the higher layers, and its weights are trained on a large dataset. We also plotted ROC curves for the different classifiers using gene expression data (Fig. 6). To assess the accuracy of our predictions, we divided the data into two sets: 75% for training and 25% for testing. The goal was to train the model on one set and evaluate its performance on another. The best results were achieved with a split of 75% training data and 25% testing data.

### XAI outcomes using SP-LIME

SP-LIME is an AI approach for explaining the predictive outcomes of Alzheimer’s medical records. The results of the XAI approach were made available to doctors and experts for better understanding and interpretation. The outcomes of interpretable approaches have shown improvements in trustworthy classifiers or predictors. We used SP-LIME to enhance the interpretability of tabular data processing. SP-LIME was employed to identify the key features of AD and evaluate the features or factors using probability values.

**Fig. 7:**
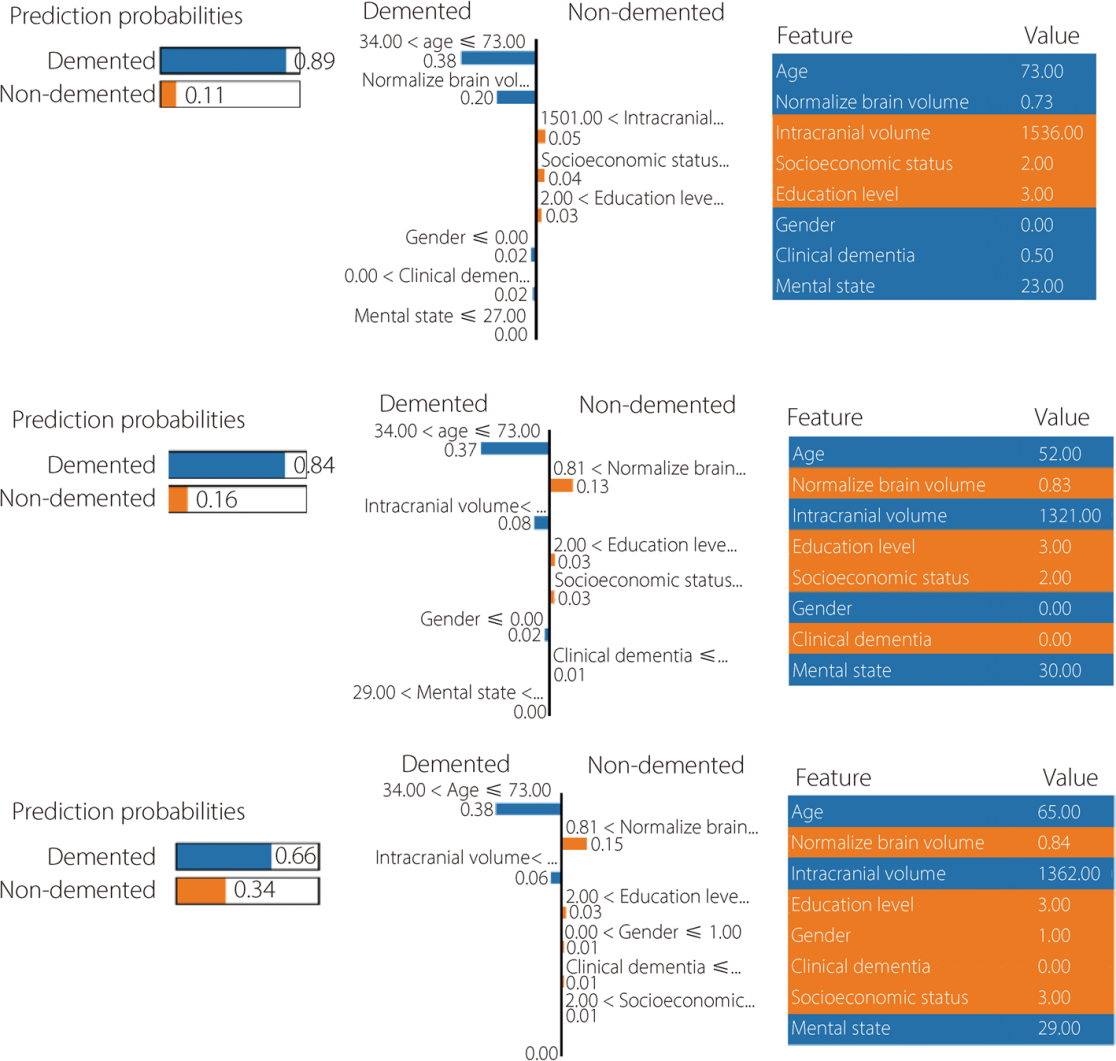
Submodular pick-LIME outcomes for the Alzheimer’s disease classification from tabular data

In the left part of Fig. 7, which represents the AD classes (demented and non-demented) based on probability values with prioritized factors, SP-LIME identifies the most important factors for satisfaction analysis. The age, normalized brain volume, and clinical dementia were highly significant factors for patients with dementia, whereas sex, mental state, and intracranial volume were highly significant factors for non-demented patients with AD, as shown in Fig. 7.

### XAI outcomes using LRP

In this subsection, we discuss the LRP outcomes for different layers. LRP outcomes help explain the layer-wise operation of a CNN for AD MRI images. These layer-wise MRI explanations interpret the prediction outcomes and provide reliable and interpretable results for doctors and experts. The LRP explains the layer operations based on a heatmap. We illustrated the convolution and pooling layers of the CNN using pixel density heat maps.

**Fig. 8:**
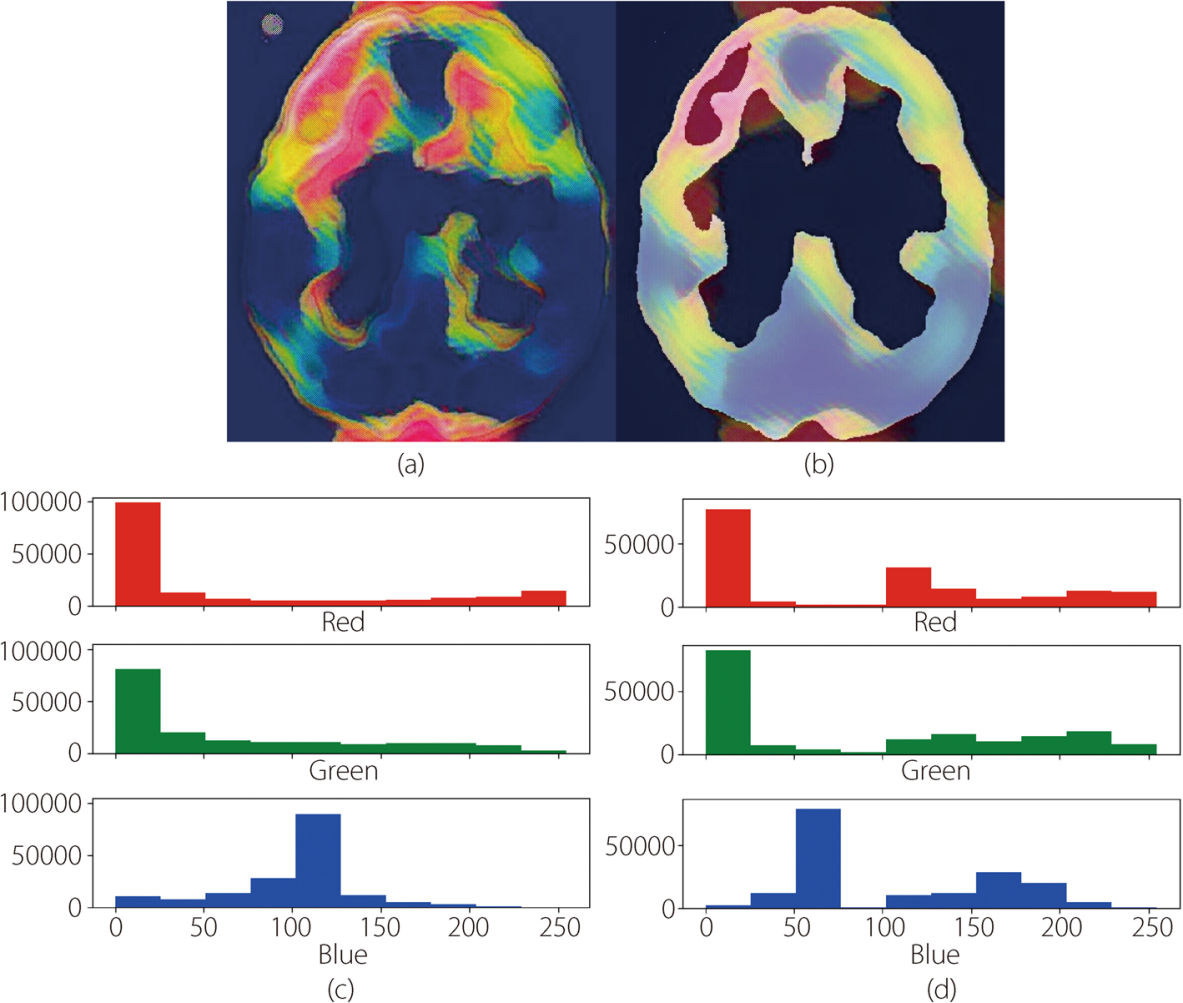
Explaining layer wise outcomes of MRI images for (**a**) convolution layer, here red color portion indicted the infected regions of the Alzheimer’s patients. (**b**) Pooling layer, where red color represents the more specific infected regions. (**c**) Pixel density heatmap analysis for three different color channels for convolution layer (**d**) pixel density heatmap analysis for pooling layer

Figure 8a shows the convolution layer outcome obtained using a heatmap. In the convolution layer, we used 24 × 24 images and generated relevance scores for the ROIs. These relevance scores were passed through the pooling layers that predicted the Alzheimer’s region. Figure 8b shows a heat map of the brain region (top-left portion). LRP explains features based on relevance scores and identifies ROIs. We also analyzed the pixel intensity using a histogram for both the convolution and pooling layer outcomes (Fig. 8c and d).

### XAI outcomes for gene expression data

**Fig. 9:**
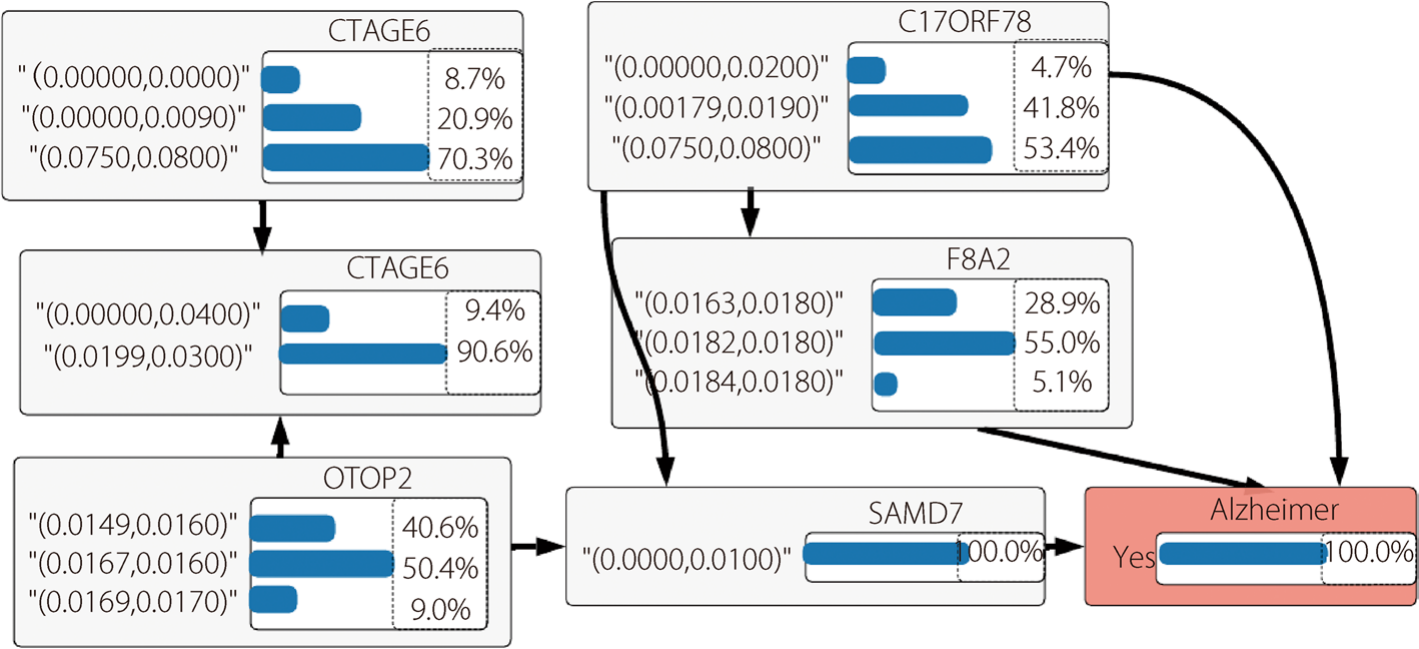
Genes associated with AD

In this subsection, we describe the explainable and interpretable processes for gene expression data. Figure 9 shows the genes that were strongly associated with AD in the test patient class. As shown in Fig. 9, based on the conditional independence, CTAGE6, F8A2, and SAMD7 were the three associated genes for prediction. CTAGE6 (CTAGE Family Member 6) is a protein-coding gene associated with gene ontology annotations and nucleotide binding. F8A2 (coagulation factor VIII-associated 2) is a protein-coding gene associated with Waisman syndrome. SAMD7 (sterile alpha motif domain containing 7) is a protein-coding gene associated with hereditary keratitis and retinitis pigmentosa.

This study offers a practical overview of interpretable methods for predicting AD with an emphasis on data diversity. This study used multimodal data from various sources including images, demographic data, and gene expression data. In this study, SP-LIME, LRP, and GGT delivered robust explanatory outcomes for multimodal AD analysis. These explanatory results will assist experts and doctors in understanding the features and genes that contribute to AD.

We evaluated the effectiveness of conventional CNN and VGG16 in identifying infected areas on MRI images. Using an ROC curve, we found that VGG16 outperformed the conventional CNN because of its deeper feature analysis (Fig. 5). We also compared the performance of using the ROC curve for different classifiers to identify patients with AD from gene expression data (Fig. 6).

We mainly emphasized the analysis of features such as age, clinical dementia, and mental status of patients with AD. Figure 7 demonstrates that age, normalized brain volume, and clinical dementia were highly significant factors for patients with dementia, whereas sex, mental state, and intracranial volume were important features for non-demented Alzheimer’s patients. In this study, XAI methods concentrated on identifying brain regions and how CNN predict AD using MRI images. Figure 8 displays the convolution layer outcome using a heat map and analyzes the pixel density for various CNN layers (Fig. 8c and d). This heatmap helps visualize brain regions that are affected by AD. The severity of the affected regions varies depending on the stage of the AD (mild, moderate, non-dementia, and very mild). XAI methods reveal biological information among patients. CTAGE6, F8A2, and SAMD7 were most significantly associated with AD (Fig. 9). Using this biological analysis, we identified important genes associated with AD and the infected areas shown on the MRI images.

## Conclusions

XAI approaches applied to multimodal data have significantly enhanced trustworthy explanations for AD analysis. Experiments were conducted using multiple types of patient data, including tabular, imaging, and gene expression data. We used the SP-LIME, LRP, and GGT approaches for reliable interpretation. SP-LIME interprets the features of AD (for example, age, mental status, and clinical dementia). LRP identifies significant brain ROIs for AD patients, which are crucial for disease severity analysis. By examining these ROIs, it is easier to understand which brain regions are responsible for specific types of ADs. Doctors and experts can readily determine the ROIs that are significant for AD. Additionally, we used GGT to identify the biology of patients with AD. These biological interpretation outcomes will help experts to understand the genes that play a substantial role in AD. Gene analysis is vital for treatment and prescription of medication.

The use of XAI methods has led to reliable and easily understandable results for doctors and researchers, empowering them to create early stage and accurate treatment plans for patients. Consequently, this contributes to a more supportive environment in society, ensuring a better diagnosis of AD for all patients, regardless of their socioeconomic background. This inclusive approach can play a crucial role in bridging healthcare disparities and promoting equitable access to quality care for patients with AD. In this study, our primary focus was on examining the interpretability and explainability of XAI methods. However, in future research, we plan to shift our attention towards investigating the counterfactual properties of these XAI methods.

## Data Availability

Not applicable.

## Abbreviations

AD: Alzheimer’s disease
MRI: Magnetic resonance imaging
R-CNN: Region-based convolutional neural network
DL: Deep learning
ML: Machine learning
XAI: Explainable artificial intelligence
LRP: Layer-wise relevance propagation
SHAP: Shapley additive explanations
SVM: Support vector machine
SP-LIME: Submodular pick local interpretable model-agnostic explanations
ROI: Regions of interest
GGT: Graphical gene tree
OASIS: Open access series of imaging studies
GNN: Graph neural network
BFS: Breadth-first search
ROC: Receiver operating characteristic
AUC: Area under the curve
AI: Artificial intelligence
CNN: Convolutional neural network
LIME: Local interpretable model-agnostic explanations
